# Assessment of the quality of life in patients with varying degrees of equalization of lower limb length discrepancy treated with Ilizarov method

**DOI:** 10.1186/s13018-021-02202-1

**Published:** 2021-01-19

**Authors:** Łukasz Pawik, Malwina Pawik, Zdzisława Wrzosek, Felicja Fink-Lwow, Piotr Morasiewicz

**Affiliations:** 1 Department of Physiotherapy of Motor Disorders and Dysfunctions, University School of Physical Education, al. Paderewskiego 35, 51-612 Wrocław, Poland; 2 Health Promotion, Faculty of Physiotherapy, University School of Physical Education, Wrocław, Poland; 3 Department of Orthopaedic and Trauma Surgery, University Hospital in Opole, Institute of Medical Sciences, University of Opole, Opole, Poland; 4 Department and Clinic of Orthopedic and Traumatological Surgery, Wrocław Medical University, Wrocław, Poland

**Keywords:** Ilizarov method, Quality of life (QoL), Limb length discrepancy (LLD)

## Abstract

**Background:**

Inequalities in leg length result in functional disorders, as they impair the biomechanics of the musculoskeletal system, significantly reducing the quality of life (QoL). This study used the WHOQoL-BREF questionnaire in patients with varying degrees of lower leg shortness who had undergone treatment by the Ilizarov method, compared to a healthy control group.

**Methods:**

Fifty-eight patients treated with the Ilizarov method for discrepancies in lower limb length were grouped by degree of limb equalization (group 1, 37 treated individuals with limb length discrepancy < 1 cm; group 2, 21 individuals with discrepancy ≥ 1 cm but not more than 4 cm). The control group 3 contained 61 healthy individuals. Patient quality of life (QoL) was assessed using a shortened version of the WHOQoL-BREF questionnaire, at least 24 months after the end of Ilizarov therapy.

**Results:**

Control subjects obtained higher scores in all domains than subjects in both treatment groups, as well as significantly higher self-assessed QoL, and health, in the physical, psychological, social, and general lifestyle domains, as compared to those with inequalities ≥ 1 cm. Furthermore, patients with inequalities ≥ 1 cm had higher odds ratios of low self-assessment (3.28 times; *p* = 0.043), low self-assessment of health (4. 09 times; *p* = 0.047), and low physical and psychological domains (respectively 6.23 times; *p* = 0.005 and 8.46 times, *p* = 0.049) compared with patients with inequality < 1 cm. The shortened version of the WHOQoL questionnaire was used.

**Conclusions:**

After at least 24 months of treatment with the Ilizarov method, patients with limb length discrepancy < 1 cm did not differ significantly from healthy individuals in the WHOQoL self-assessment of mental functioning, social, or life satisfaction.

## Background

Limb length inequality is a relatively common impairment and poses a significant challenge for contemporary orthopedics and biomechanics. If the pathology affects upper extremities, it has only a cosmetic aspect; however, in the case of asymmetry of the lower limb (LL), it is also a functional problem, impairing the functioning of the limb and the biomechanics of the musculoskeletal system, and significantly reducing the quality of life (QoL) of affected patients [1–9].

In the case of LL inequality greater than 2.5 cm, the preferred choice for limb equalization is a lengthening of the shorter limb using the Ilizarov method. This method of treatment, with its optional correction of coexisting axial deformations, is one of only two ways of correcting moderate to severe limb-length discrepancies, apart from stimulating epiphyseal plate activity [3, 4, 10, 11].

The main reason for surgery is to improve limb functioning and to eliminate the consequences of this pathology in the musculoskeletal system. Correct balance and the ability to walk without pain allow proper functioning and physical activity and thus significantly improve the QoL of patients [12–17].

Orthopedic surgery aiming to eliminate limb-length discrepancies does not succeed in all patients: some experience a reduction in joint mobility as a result of equalization. Blood supply disorders or neurological disorders may also occur. Both of those conditions are indications to urgently discontinue, or even to partial retract, equalization in order to avoid complications. If there are comorbidities such as paresis or a significant restriction of mobility at the hip or knee joints, it is advisable to leave a limb length discrepancy of 1–3 cm in order to achieve proper limb function. Full limb-length equalization in such cases can disturb the biomechanics and hamper the compensating functions of the musculoskeletal system. If this happens, the functioning of the equalized limb, and as a result the patients’ QoL, may prove unsatisfactory.

The literature contains reports where the patient’s pain or subjective satisfaction with the treatment are evaluated [18–23]. However, these studies examine the patients’ satisfaction immediately after removing the apparatus, and they also fail to specify how the degree of limb-length equalization—or the lack thereof—affect the QoL of patients in the long term.

In our opinion, such analysis is justified: clinical experience shows that, following comprehensive treatment where the physician believes that the limb length correction has succeeded, the QoL of patients still often appears to be unsatisfactory.

The aim of this study was to assess the QoL of patients with varying degrees of equalization of limb length discrepancy, within the shank area, at least after 24 months treatment with the Ilizarov method, and to compare this to the control healthy group of individuals with equal LL length.

## Methods

The study was approved by the local bioethics commission (case no. KB-585/2011). All subjects gave their informed consent to participate in the study and to answer the questionnaire. The study was conducted in accordance with the Code of Ethics of the World Medical Association (Declaration of Helsinki) for experiments involving humans. The study was retrospective. It was conducted between 2012 and 2018. In order to achieve research objectives, 119 people were included.

The patient group contained 25 women and 33 men aged 22.3 ± 2.21, with a (5–95) median BMI value of 24.29 kg/m^2^ (range 20.54–28.03 kg/m^2^), who were treated with the Ilizarov method due to LL length discrepancy in the lower leg. Two subgroups were distinguished on the basis of on the degree of limb equalization. The number of months after surgery did not differ significantly between the two groups of patients, at 40.11 ± 12.2 months vs. 42.0 ± 11.2 months (*P* = 0.106).

All patients underwent equalization of a lower limb due to a length discrepancy of over 2.5 cm, or over 1 cm, with concomitant deformation requiring surgical correction. All the patients had isolated shortness of the tibia.

The Ilizarov fixator used in the study consisted of 3 rings assembled on the shank with Kirschner wires with a diameter of 1.8 mm. After fitting the stabilizer, the tibia and fibula were drilled radially at the proximal epiphysis, and corticotomy was carried out through the 1–2-cm skin incision. The lengthening was halted and equalization not performed in patients with complications (limitations in joint mobility, neurological disorders, or vascular disorders) and in those who, apart from limb shortness, were also diagnosed with a primary limitation of joint mobility of the limb or other concomitant pathologies (such as muscle weakness, instability) before treatment.

Bone grafting was performed in each of the patients. The intensity of pain was not assessed during the present study.

The healthy control group consisted of 61 people of both sexes with no inequality, functional disorder, or pain in the lower extremities. The control group and the study groups did not differ statistically significantly in body height, gender, or BMI. The control group consisted of adults referred by a physician or orthopedist as otherwise healthy patients without any dysfunctions within the osteoarticular system. All subjects gave their informed consent to participate in the study. An orthoroentgenogram was used to measure the limb length.

Group 1 consisted of 37 subjects (16 women, 21 men) who had undergone surgery, whose limb length difference was less than 1 cm and who had not been subjected to further correction, because it fell within physiological asymmetry. The differences in LL before and after Ilizarov treatment in group 1 was 20 mm (range 8.8–37.9 mm) vs. 0 mm (range 0–5.2 mm) (*p* < 0.001).

Group 2 consisted of 21 subjects (9 women, 12 men) who had undergone surgery and whose remaining limb length deficit 1 cm or more, but less than 4 cm. Such length deficits of 1–4 cm remained due to complications (joint mobility limitation, neurological disorders, vascular disorders) that prevented the full equalization of the limb. In some patients in group 2, in the doctor’s opinion, the shortness did not require correction, as the inequality was well within the range of compensation. In such patients, apart from shortness, primary limitations like joint mobility of the limb or other concomitant pathologies (such as muscle weakness or instability) were diagnosed before treatment and were considered as indications for leaving a residual shortness of 1–3 cm. The differences in LL before and after Ilizarov treatment in group 2 was 38 mm (23.0–48.7 mm) vs. 13.0 mm (11.0–18.0 mm) (*p* < 0.001).

Group 3, the control group, consisted of 61 healthy individuals (29 women, 32 men; aged 21.9 ± 1.9), in whom there was no dysfunction or pain in their medical history, and there was no LL length inequality.

**Fig. 1:**
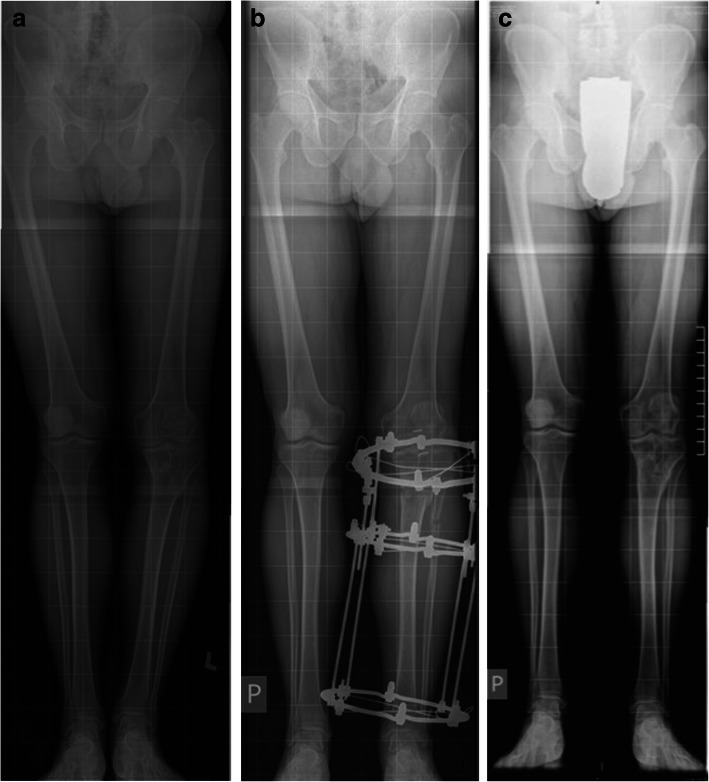
Computed radiography in a standing position. **a** Patient before treatment. **b** Patient during the treatment process. **c** Patient after treatment

The inclusion criteria were that they had given their informed consent to participate; that their full medical and radiological records were available; that they had completed treatment with the Ilizarov method in the shank area; that their limb length deficit after Ilizarov treatment was less than 4 cm; that they had no limb axis disorders that required further correction; and that they had undergone an observation period of at least 6 months. Qualification to the study was based on an analysis of medical records, physical examination, and computed radiography carried out in the standing position (Fig. 1). This method is considered one of the best options for measuring LL length; it has a maximum error of 2 mm. It is safe and recommended by many researchers as a method that allows the patient’s LLs to be assessed even more comprehensively [24–26].

The program enabled the researchers to work and to measure the length of the lower extremities, setting a line from the highest point of the femoral head to the point located at the mid-width of the articular surface of the distal epiphysis of the tibia.

Patient QoL was assessed out using the World Health Organization Quality of Life Test-BREF (WHOQoL-BREF) questionnaire. This universal research tool is used for subjective assessment of QoL in healthy individuals, in people with ailing health, and in those undergoing various treatments [27]. The short version of the questionnaire includes 26 questions on four main areas:

- Physical functioning: assessment of the activities of daily living, mobility, pain, ability to work, rest, sleep, fatigue and energy levels, and reliance on treatment or pharmacotherapy
- Mental functioning: assessment of positive and negative emotions, visual appearance, spirituality, faith and religion, and the ability to concentrate and learn
- Social relations: assessment of social and personal relationships and sexual activity
- General lifestyle: assessment of satisfaction with life, work, home environment, financial resources, freedom, recreation, physical and mental state, and the immediate environment [28]

Two additional questions in the questionnaire concerned the assessment of overall QoL and self-assessment of health [26]. Subjects responded to 26 questions, choosing one of five ranked responses. The higher the numerical result of the overall assessment of life quality, self-assessment of health, and individual aspects of life, the more favorable the self-assessment was in respect to particular domains of the QoL.

### Statistical analysis

The data were analyzed using the SigmaPlot statistics package, version 13 (Systat Software, London, UK). The continuous variables were first analyzed for normal distribution using the Kolmogorov–Smirnoff test with the Lilliefors correction test. All values are expressed as means ± standard deviations (SDs) or as medians with 95% confidence intervals (Cis). Not all data passed the normality test, so the significance of the differences between two groups were analyzed using the Mann–Whitney *U* test. The significance of differences between three groups was tested using the Kruskal–Wallis one-way analysis of variance on ranks (ANOVA on ranks).

As groups of patients varied in size, all pairwise multiple comparisons were performed using Dunn’s method. Spearman correlation analysis was used to test an association between QoL and months since surgery or limb length inequality by sex. Multiple logistic regression was employed to estimate the risk of a reduction in QoL as reported by the WHOQoL-BREF questionnaire associated with limb length inequality level (LL < 1 cm vs. LL ≥ 1 cm), overweight (BMI < 25 kg/m^2^ vs BMI ≥ 25 kg/m^2^), and sex. To estimate the odds ratios, the data were transformed into dummy variables using reference coding. The details of the coding are shown in the legend of Table 3. A *P* value < 0.05 was considered statistically significant.

## Results

**Fig. 2:**
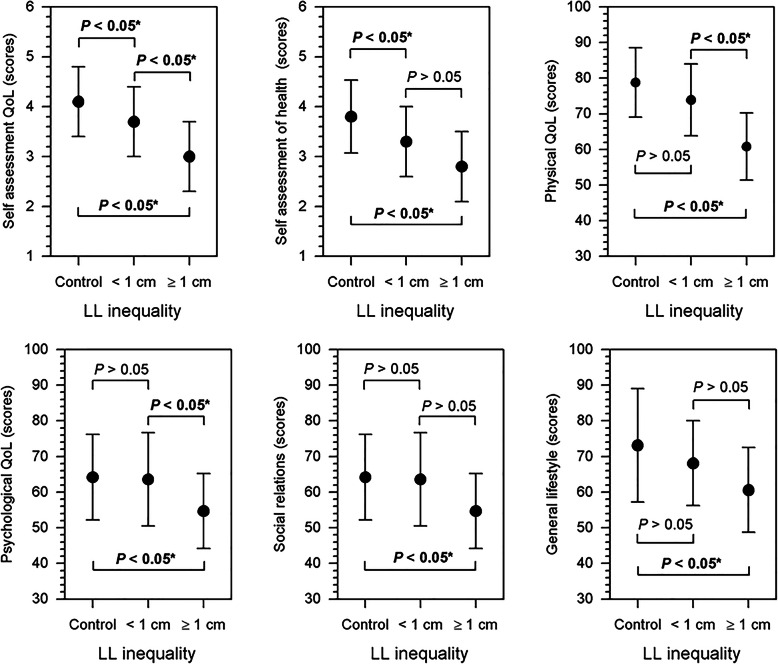
The quality of life of patients after treatment with the Ilizarov method. LL, lower limb length; QoL, quality of life

Limb-length discrepancy prior to surgery statistically significantly differentiated both patient groups. In patients of group 1, who had post-treatment limb length inequalities of up to 1 cm, the difference was 20.95 ± 10.61 mm, while in group 2 (patients with LL shortness ≥ 1 cm), this was 36.81 ± 7.46 mm (*p* < 0.001). The mean values for LL shortness also differed significantly between group 1 and group 2 after Ilizarov treatment (*p* < 0.001), at 1.11 ± 1.03 mm vs. 13.86 ± 2.33 mm. In both groups, the percentage of patients with pin site infection was similar, amounting to about 30%. The average axis correction in both groups was also similar at 12°. A comparison of assessment scores of QoL domains between the two patient groups and controls is shown on Fig. 2.

Figure 2 shows a comparison of WHOQoL-BREF scores for individual QoL domains between the three groups: the two patient groups (with inequalities in LL < 1 cm and LL ≥ 1 cm) and the controls. Those in the control group scored highest for individual perception of QoL, overall perception of their health, and all four QoL domains (physical, psychological, social relations, and general lifestyle). The more points scored (and the higher the mean) in a given area, the better the QoL, and subjects in the control group obtained the highest scores in all domains. A similar trend was seen when comparing the two patient groups, where higher QoL in all components was seen for individuals with a smaller discrepancy (LL < 1cm) (Fig. 2). In summary, the control subjects scored highest in all domains, but scored statistically significantly higher than both treatment groups for self-assessment QoL and assessment of health, and higher than treatment group 2 in the physical, psychological, social, and general lifestyle domains (*p* < 0.05) (Fig. 2). Comparison of groups 1 and 2 demonstrated significantly higher QoL in the self-assessment, physical, and psychological domains in patients with LLD < 1 cm.

This means that patients who had unequal LLD < 1 cm after Ilizarov treatment assessed their mental and social functioning, as well as their satisfaction with life, work, and their physical and mental state in a similar way to those who did not exhibit any LL length discrepancies or dysfunctions (the healthy individuals). It is worth noting that, in the questions concerning QoL and health, the treatment groups did not attain the mean scores scored by the healthy control subjects. The statistically significant mean score values vary not only in relation to the control group, but also between the treatment groups. This demonstrates that, after LL equalization, even when only a slight limb length discrepancy remains (< 1 cm), subjects assess their QoL and their health less favorably than those in the control group.

The associations between domains of QoL by WHOQoL-BREF and time after surgery are shown in Table 1.

**Table 1:** Spearman rank correlation coefficients for the association between domains of QoL and time after surgery [months] in the two patient groups differing in lower limb length inequality

QoL domain	LL shortness < 1 cm (*N* = 37)		LL shortness ≥ 1 cm (*N* = 21)	
	*r*	*P*	*r*	*P*
Self-assessment of QoL	0.079	0.640	0.382	0.086
Self-assessment of health	0.060	0.722	0.351	0.117
Physical	0.159	0.344	**0.476**	0.029*
Psychological	− 0.006	0.973	0.353	0.114
Social relations	0.252	0.131	0.409	0.0645
General lifestyle	0.059	0.728	**0.552**	0.010*

*LL* lower limb length, *QoL* quality of life, *r* correlation coefficient, *P* statistical significance

*indicates statistically significant increased odds ratio

We observed significantly positive associations in physical and general lifestyle domains but only in patients with LL ≥ 1 cm (*P* < 0.029; *P* < 0.010 respectively). We did not observe any significant associations in patients with shorter differences. This shows that people without fully equalized LLDs only report improvement in these two domains of QoL in the long-term after Ilizarov treatment. The associations between LL and QoL in patient groups by sex are presented in Table 2. We observed significantly negative associations between LL and QoL for all domains, independent of sex.

**Table 2:** Spearman rank correlation coefficients for the association between domains of QoL and lower limb length inequality (LL) in patient groups, by sex

QoL domain	Women *N* = 25		Men *N* = 33	
	*r*	*P*	*r*	*P*
Self-assessment of QoL	**− 0.440**	0.027*	**− 0.568**	< 0.001*
Self-assessment of health	**− 0.598**	0.002*	**− 0.497**	0.003*
Physical	**− 0.613**	0.001*	**− 0.647**	< 0.001*
Psychological	**− 0.512**	0.009*	**− 0.592**	< 0.001*
Social relations	**− 0.489**	0.013*	**− 0.481**	0.005*
General lifestyle	**− 0.550**	0.005*	**− 0.439**	0.011*

*QoL* quality of life, *r* correlation coefficient, *P* statistical significance

*indicates statistically significant increased odds ratio

Table 3 presents a logistic regression analysis, where the odds ratios (ORs) of reduced QoL are assessed by greater inequality level (LL), overweight, and sex. Patients with inequality LLD ≥ 1 presented higher odds ratios of low self-assessment of QoL (3.28 times; *p* = 0.043), low self-assessment of health (4.09 times; *p* = 0.047), and low physical and psychological domains (respectively 6.23 times; *p* = 0.005 and 8.46 times, *p* = 0.049) than patients with inequality LLD < 1, after more than 24 months (meantime forty) of Ilizarov treatment (Table 3). We did not find a significant link between sex and any domain of QoL. Furthermore, we observed a significantly higher odds ratio of reduced self-assessment of quality of life in overweight patients than in those with normal BMI (3.05 times, *p* = 0.05). When we take into account patients with both LLD ≥ 1 cm and overweight, there is a significant relationship with the self-assessment of quality of life and physical domain. This means that LLD ≥ 1 and BMI ≥ 25 kg/m^2^ reduced the self-assessment of quality of life by a factor of 4.4 (*p* = 0.039); and in the physical domain by 7.2 (*p* = 0.016).

**Table 3:** Odds ratios for reduced quality of life as estimated by WHOQoL-BREF in patients with different grades of limb length inequality who underwent Ilizarov treatment

	Odds ratios (95% CI), *p*	
	**Self-assessment of quality of life (scores < 4)**	**Self-assessment of health (scores < 4)**
Limb length inequality (≥ 1 cm)	**3.28 (1.04–10.35) 0.043**^**a**^	**4.09 (1.02–16.38) 0.047**^**a**^
Sex	0.68 (0.24–1.93) 0.470	0.67 (0.22–2.04) 0.478
Overweight^b^ (BMI ≥ 25 kg/m^2^)	**3.05 (1.00–9.27) 0.050**^**a**^	3.17 (0.89–11.30) 0.076
Limb inequality ≥ 1 cm + overweight	**4.40 (1.08–7.98) 0.039**^**a**^	8.18 (0.18–68.4) 0.052
	**Physical domain (scores < 75)**	**Psychological domain (scores < 75)**
Limb length inequality (≥ 1 cm)	**6.23 (1.75–22.23) 0.005**^**a**^	**8.46 (1.00–71.10) 0.049**^**a**^
Sex	1.06 (0.37–3.02) 0.912	1.08 (0.30–3.90) 0.910
Overweight^b^ (BMI ≥ 25 kg/m^2^)	1.47 (0.51–4.27) 0.480	2.31 (0.55–9.65) 0.252
Limb inequality ≥ 1 cm + overweight	**7.20 (1.44–36.03) 0.016**^**a**^	4.33 (0.51–37.03) 0.180
	**Social relations (scores < 75)**	**General lifestyle (scores < 75)**
Limb length inequality (≥ 1 cm)	2.59 (0.72–9.27) 0.144	0.96 (0.30–3.10) 0.951
Sex	1.29 (0.41–4.00) 0.664	1.10 (0.59–2.05) 0.760
Overweight^b^ (BMI ≥ 25 kg/m^2^)	1.48 (0.46–4.73) 0.510	1.13 (0.35–3.71) 0.836
Limb inequality ≥ 1 cm + overweight	1.90 (0.46–7.85) 0.377	2.80 (0.55–14.23) 0.215

Reference coding: limb length inequality ≥ 1 cm, 1; sex: female, 1; overweight, 1

*CI* confidence intervals

^a^Bold typeface indicates statistically significant increased odds ratio

^b^None of the treated patients was obese

## Discussion

Among those individuals treated for LL length discrepancy with the Ilizarov method, there is a belief that the restoration of anatomical limb length and axis is equivalent to the restoration of its function, which thus has an indirect effect on QoL. Most authors who have looked at functional outcomes of locomotor limb-length equalization point to the improvements in QoL in the four domains, and to the increments in self-assessment scores of QoL and health in all treated patients [9, 18, 19, 29, 30]. In the present study, however, we demonstrated that the mean QoL scores in particular areas in the two groups treated with the Ilizarov method are lower than in healthy individuals. We could not, however, establish statistically significant differences in the case of psychological, general lifestyle, or social functioning of people with limb shortness less than 1 cm, as compared to the control group, after more than 24 months (meantime forty month) of treatment with the Ilizarov method. Many authors have performed subjective assessments of health and QoL in patients following treatment with the Ilizarov method.

Prevalence of pain, subjective satisfaction with results, and appearance of the limb after Ilizarov treatment were described [18–23]. All studies, similarly to us, reported improvements in various aspects of QoL after treatment. However, most other studies only examined patients immediately after dismantling the Ilizarov apparatus. Little research has examined how the degree of limb-length equalization influenced the QoL in the long term after the surgical intervention. In our research, the mean time from the period of surgery was 40.11 months for the group with an LL shortness of less than 1 cm and 42.05 months for the group with an LL shortness of 1 cm or more. This is a sufficient period of time for patients to rationally evaluate the effects of their treatment and to assess the improvements to particular domains of their QoL.

In the study of Ramaker et al. on a group of 26 patients treated with the distraction osteogenesis method, no effect on QoL was observed as a result of the LL inequality remaining after treatment, as examined by a Dutch questionnaire on mental health and depression in children. QoL was nonetheless evaluated 16–67 months after the first surgery [22]. Different conclusions were reached by Vitale et al., who studied a group of 76 people with LL length asymmetry [17]. The aim of their study was to determine the effects of equalization of limb length discrepancy on the QoL of patients using the Child Health Questionnaire. In line with their expectations, patients with a limb shortness of up to 2 cm had far better results for QoL, especially in the psychosocial domain, than did patients in the group with length asymmetry over 2 cm. In explaining these results, the authors concluded that it resulted from different levels of self-esteem. Individuals with locomotive limb length shortness greater than 2 cm often face limitations in their daily lives, which principally relate to physical activity.

Similar conclusions were reached by Moraal et al., who examined the long-term results of treating patients with the Ilizarov method [29]. Assessing a group of 37 patients before surgery and for an average period of 7 years after surgery, they found improvements in their health and in their everyday life functioning. In patients where unequal LL length remained and was greater than 2 cm, lower scores were observed, particularly in the areas of pain, sleep disorders, and psychosocial functioning and relations. Limb shortness greater than 2 cm was found to significantly lower the QoL of patients treated with Ilizarov method.

In our study, the mean scores for particular QoL domains were higher in patients with a limb shortness of up to 1 cm than in those with an unequal limb length greater than 1 cm. This means that the equalization of limb length discrepancy has a significant effect on the QoL of patients treated with the Ilizarov method. Comparing the individual QoL domains, it was found that patients whose limb length discrepancy after correction was less than 1 cm assessed their mental and social functioning and life satisfaction in a similar way to people who had never had an inequality or LL dysfunction. We can thus assume that equalization of LL brings not only improvements to limb functioning, but also to psychosocial and general aspects of lifestyle.

It is also worth noting that the only exception was patients’ subjective assessment of QoL and health. In this case, all patients treated with the distraction osteogenesis method had lower scores than those in the control group.

QoL has a multifactorial ground. In our work, we take into account not only the LL inequality but also overweight. A study of a few European countries has shown an association between the level of obesity and individual quality of life, mainly related to the concomitant reduction in physical and mental well-being [31]. In our work, overweight significantly reduced self-assessed QoL by a factor of over 3 for overweight patient, compared to normal BMI (body mass index). We found that greater inequality and additional overweight were associated with lower QoL, particularly in the self-assessment quality of life and physical domains. Other researchers noted that congenital LL length discrepancy and greater difference in limb length contributed to greater number of complications and longer healing process [32]. Both those factors in addition to obesity may reduce QoL as well. The result from our study and data from the literature can serve as a basis for promoting the maintenance of proper BMI in such people though a health-promoting lifestyle, particularly one including regular physical activity. We might suspect that patients with a low level of physical activity as a result of the dysfunction and of the limitations they have experience in developing health-promoting behaviors related to physically active leisure time, prior to the elimination of LLD. Regular leisure-time physical activity tailored to each patient is a beneficial factor for somatic health and prevents lifestyle diseases [33], and also improves QoL [34, 35].

Summarizing our results, we concluded that treatment using the Ilizarov apparatus is complex and does not always give full equalization of limb length. Despite improvements in surgical techniques, the external fixators, and the individualized rehabilitations, the negative impact of being left with a locomotor limb length shortness of more than 1 cm after surgery evidently affects the assessment of QoL. Despite the fact that, such result is satisfactory from a medical point of view, it should be noted that such asymmetry of limb length significantly affects the QoL of patients, particularly with regard to mental functioning and social relationships. This fact speaks to the need for therapy and treatment of the patient as a whole.

When assessing and analyzing the QoL of patients in each treatment group, it should be stated that any remaining discrepancy should be less than 1 cm. This makes it possible to minimize the risk of complications and, over the longer term, to normalize the individual domains of patients’ QoL.

This research was planned and executed as a retrospective study. We envisage that future research will consider the situation before and after the lengthening of the shorter extremity with the Ilizarov method. This would allow us to more accurately determine the impact of limb-length equalization on individual QoL domains of patients treated with the Ilizarov apparatus. Our results here should be treated as preliminary, given the small size of the group. We wish to stress that reaching a wider group of subjects within a period that is some distance in time from end treatment is difficult, due to the limited participation of patients and small number of patients treated with the Ilizarov method. On the other hand, the study group was ethnically and culturally homogeneous, eliminating the additional factors that might affect QoL

Limitations

This study has several limitations. Possible errors in limb length measurement may have occurred during radiological evaluation of the patients, especially when measuring inequalities below 1 cm (the measurement error was 2 mm). Patient groups were also relatively small, and the intensity of pain and the range of joint movement were not evaluated. Furthermore, due to the specific nature of the treatment, patients were not randomly assigned to the study groups, and the researchers were not blinded. However, as the conclusions of the study were interesting, so we plan to address the limitations in our future research by evaluating QoL in a larger group of patients and considering the intensity of pain and range of joint motion.

## Conclusions

1. Patients with limb length discrepancies below 1 cm, after meantime 40 months of Ilizarov treatment, did not differ significantly from healthy individuals in the WHO-QoL self-assessment of mental functioning, social, or life satisfaction.
2. Higher grades of inequality were associated with lower QoL, particularly in the domains of low self-assessment, self-assessment of health, or physical and psychological domains in patients treated with the Ilizarov method.

## Data Availability

The datasets used and analyzed in this study are available from the corresponding author on reasonable request.

## Abbreviations

BMI: Body mass index
LL: Lower limb
QoL: Quality of life
WHOQoL-BREF: World Health Organization Quality of Life Test short version
